# Reliability of pre-admission patient-reported outcome measures postoperatively assessed via proxies: a prospective, multicenter observational study

**DOI:** 10.1186/s13054-025-05431-6

**Published:** 2025-05-19

**Authors:** Julius J. Grunow, Leonie Hartmann, Bernhard Ulm, Mathilde Maechler, Manfred Blobner, Katharina Seidenspinner, Lukas Schoennagel, Steffen Weber-Carstens, Kristina Fuest, Tobias Wollersheim, Stefan J. Schaller

**Affiliations:** 1 Charité-Universitätsmedizin Berlin, Corporate Member of Freie Universität Berlin and Humboldt Universität zu Berlin, Department of Anaesthesiology and Intensive Care Medicine (CCM/CVK), Charitéplatz 1, 10117 Berlin, Germany; 2 Technical University of Munich, School of Medicine and Health, Klinikum rechts der Isar, Department of Anaesthesiology and Intensive Care Medicine, Munich, Germany; 3 University of Ulm, Faculty of Medicine, Department of Anesthesiology and Intensive Care Medicine, Ulm, Germany; 4 Department of Cardiology, Vivantes Klinikum im Friedrichshain, Berlin, Germany; 5 Medical University of Vienna, Department of Anaesthesia, Intensive Care Medicine and Pain Medicine, Clinical Division of General Anaesthesia and Intensive Care Medicine, Spitalgasse 23, 1090 Vienna, Austria

**Keywords:** Patient-reported outcome measures, Pre-admission status, Critical care, Critical illness, Proxy assessment, Health-related quality of life, Physical function, Cognitive function

## Abstract

**Background:**

Pre-admission status obtained through patient-reported outcome measures is an essential metric in both clinical and research settings for prognostication and treatment decisions. It is frequently collected by proxies, although its reliability has yet to be thoroughly investigated. The objective was to determine the reliability of proxy assessments regarding pre-ICU admission status via patient-reported outcome measures and to explore the impact of the ICU setting on these assessments.

**Methods:**

Prospective multicentre observational study in two tertiary care university hospitals in Germany, including surgical adult patients able to independently answer the patient-reported outcome measures (SF-36, EQ-5D-5L, WHODAS 2.0, IADL, and Barthel Index) with a proxy available. Patients were interviewed pre-operatively, while proxies were interviewed post-operatively in the ICU or normal ward, depending on the patient's location. The reliability of patient-reported outcome measures was analyzed using Bland–Altman plots and Cohen’s kappa.

**Results:**

Of 204 patient-proxy pairs, 102 were admitted to an ICU. The median patient and proxy age were 69 and 64 years, with 41% and 68% female, respectively. Bland–Altman plots demonstrated insufficient reliability of proxy ratings, as the 95% limits of agreement fell outside the minimal clinically important difference (MCID) for all questionnaires. However, a significant bias was evident only among ICU patients, showing worse ratings from proxies for the WHODAS 2.0, IADL, and subsets of the SF-36. For the EQ-5D, bias appeared in both the ICU and non-ICU cohorts. The dichotomous analysis of the within-pairs-difference supported the findings, revealing a high proportion of pairs with differences outside the MCID (n (%)—SF-36 PCS normal ward: 47 (46%), ICU: 58 (57%); SF-36 MCS normal ward: 59 (58%), ICU: 62 (61%); WHODAS 2.0 normal ward: 58 (57%), ICU: 78 (77%); EQ-5D-5L normal ward: 40 (39%), ICU: 46 (45%); Barthel-Index normal ward: 22 (22%), ICU: 21 (21%)). Cohen’s kappa indicated moderate reliability for the IADL.

**Conclusion:**

The reliability of proxy assessment with the instruments used was insufficient, exhibiting a significant bias in the pre-admission status of ICU patients; therefore, it should be applied with caution.

**Trial registration**: ClinicalTrials.gov (NCT03785444—28th of December 2018).

**Graphical abstract:**

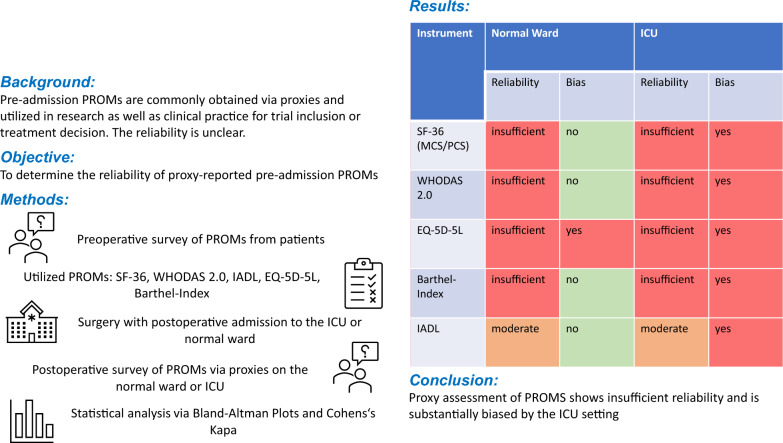

**Supplementary Information:**

The online version contains supplementary material available at 10.1186/s13054-025-05431-6.

## Introduction

Patients'physical, mental and cognitive status before intensive care is a critical factor influencing the course of treatment and its outcome, especially in an ageing population [1, 2]. In particular, the pre-admission status is an independent and robust predictor for intensive care unit (ICU) and hospital mortality [3, 4]. Additionally, pre-admission status affects morbidity, as it has been shown to impact dependency, quality of life, and readmission rates after discharge [5]. Therefore, it is logical—albeit ethically debated—that the inclusion of pre-admission status in triage tools has been proposed in situations of ICU bed shortages, such as during the COVID-19 pandemic [6]. Typically, pre-admission status is assessed through various patient-reported outcome measures (PROM), which demonstrate a strong correlation with actual performance status following discharge from the ICU [7].

Unfortunately, the pre-admission status of patients in ICUs is often unknown due to the frequently unexpected onset of critical illness. Therefore, it was proposed that the missing information on pre-admission status be obtained through proxy assessment [8]. However, the proxy assessment had an inherent bias in a propensity score-matched analysis of community-dwelling Medicare beneficiaries [9]. Similarly, a propensity score-matched cohort, consisting of more than 22,000 participants selected from the general population in a national health survey in China, demonstrated a systematic bias wherein proxies undervalued the health status [10]. A systematic review concluded that the quality of proxy assessment was heterogeneous and depended on the instrument and the setting [11]. Consequently, it is challenging to extrapolate results to different cohorts and outcome measures. Earlier studies suggested insufficient reliability in the ICU cohort due to multiple biases. The trial by Rogers et al. gathered patient and proxy responses at ICU discharge, thus eliminating the confounder that might impact proxy response when the patient's outcome is unclear. Hofhuis et al. included only patients able to respond at ICU admission, rendering proxy assessment redundant. Hence, the evidence remains inconclusive [12, 13]. Furthermore, there is a lack of studies investigating the impact of the ICU setting on proxy assessment.

Although the reliability of ICU proxy assessment of PROMs is uncertain, it is widely utilized in clinical settings for prognostication and treatment decisions, as well as in research for study inclusion. This study aimed to prospectively investigate the reliability of proxy assessments concerning pre-ICU admission status acquired through PROMs and to demonstrate the influence of the ICU setting on the proxy assessment.

## Methods

This prospective two-centre observational study was approved by the institutional ethics committee of the medical faculty of the Technical University of Munich (542/18S) and conducted at Charité-Universitätsmedizin Berlin and Klinikum rechts der Isar of the Technical University of Munich, following the Declaration of Helsinki. It was registered at ClinicalTrials.gov (NCT03785444) on December 28th, 2018.

### Participants

Patients were eligible for recruitment if they were at least 18 years old and a proxy was able to be present during the post-operative period. A proxy was seen as a person that is emotionally or socially connected to the patient. A familial or legal tie was not a requirement in order to reflect the modern and diverse environment of social relationships. The inability of the patient to answer the PROMs independently before the operation was an exclusion criterion. Patients and proxies were enrolled in the study after obtaining written informed consent. Screening was conducted using a convenient sampling method.

### Data collection

Data was collected by trained and dedicated study staff after enrolment to accurately represent the scenario in which proxies are asked to respond when patients cannot. Consequently, patients were interviewed preoperatively in the normal ward or during the pre-anaesthesia consultation. As a result, proxies were interviewed postoperatively, i.e., when patients would be non-responsive, either in the normal ward, in the ICU, or by telephone. This depended mainly on the availability of the proxy, mirroring a real-world clinical scenario. The responses were provided by both patients and proxies in written form or verbally, and the interviewer documented them in the case report form. The interview covered baseline demographic variables and all the items from the different PROMs. The interview duration depended on the respondent and was not a priori set or limited.

### Variables

Demographic data was recorded for the patients (i.e., age, sex, hospital length of stay, pre-existing medical conditions, pre-existing medication for those illnesses, number of prior surgeries as well as surgical discipline for the upcoming surgery) and proxies (i.e., age and sex), as also collected, along with qualitative and quantitative values regarding their relationships, including relationship status, whether they share a household, and how often they visit, call, or text. Various validated patient-reported outcome measures (PROMs), commonly used in studies investigating long-term outcomes of critical illness and recommended by multiple core outcome sets, were employed to assess the patients’ current status [14, 15]. Health-related quality of life was evaluated using the Medical Outcome Study Short-Form Health Survey 36 (SF-36), as recommended by the core outcome sets for acute respiratory failure, along with nutritional and metabolic interventions. Overall disability and health were assessed with the World Health Organization Disability Assessment Schedule 2.0 (WHODAS 2.0), due to its strong correlation with performance-based outcome measures, as well as the EQ-5D-5L, which is recommended by the core outcome set for respiratory failure. Independence in daily living was measured using the instrumental activities of daily living (IADL) scale and the Barthel index, as advised by the core outcome set for nutritional and metabolic interventions, with the modification that these two measures were to be reported for the situation two weeks prior to the current hospital admission [7, 16–22].

### Sample size

The results of patient versus proxies were compared using Bland-Altman diagrams. For that, a test size of 100 patients has been recommended [23]. This resulted in 200 patients (100 ICU patients and 100 non-ICU patients).

### Statistical analysis

For the SF-36, the Mental and Physical Component Score (MCS/PCS) were calculated, and missing data was handled according to the manual by Ware et al. [16, 17]. The sum of the 12 items from the WHODAS 2.0 was calculated and converted to a percentage, as previously done by Hodgson et al. and Shulman et al.; missing data were handled as outlined by the manual [24–26]. The index score for the EQ-5D-5L was calculated according to the German value set [27]. IADL and Barthel sum scores were calculated as described in the initial publication [20, 22].

Missing items in the scores were imputed using a classification and regression tree. Following this, patient scores were calculated, incorporating both reported and imputed items. A sensitivity analysis excluding imputed values is provided in the supplement.

Descriptive statistics are presented for continuous variables based on the evaluation of normality of distribution using the Shapiro–Wilk test and reported as either median (interquartile range—IQR) or mean (standard deviation—SD). Categorical variables are presented as counts (percentages). Differences between groups were assessed similarly with either a t-test or Mann–Whitney U test for continuous variables, depending on the results of the Shapiro–Wilk test, and using the Chi-Square test for categorical variables.

Interrater reliability for continuous variables was evaluated via Bland–Altman plots and unweighted and weighted Cohen’s kappa for categorical variables. The clinical acceptability of the deviation was assessed by comparing the 95%-limits of agreement to the minimal clinically important difference (MCID) for the respective questionnaire (5 for the SF-36 PCS, 5.5 for the MCS, 5 for the WHODAS 2.0, 0.18 for the EQ-5D and 9.8 for the Barthel Index) [26, 28–30]. The rationale behind this comparison is that if the deviation between patients and proxies is larger than the MCID a clinically significant difference between two assessments could be caused only by the difference in responder. A systematic bias was evaluated via the 95% confidence interval of the mean difference. The difference was derived by subtracting the proxy score from the patient scores. This difference was also analyzed dichotomously with the MCID as the cut-off value. Statistical analysis was performed with IBM SPSS Statistics for Macintosh, Version 27.0 (IBM Corp, Armonk, NY, USA) and R Foundation for Statistical Computing (Vienna, Austria). Figures were created using GraphPad Prism version 7.0.0 for Macintosh (GraphPad Software, San Diego, CA, USA) and R Foundation for Statistical Computing (Vienna, Austria).

## Results

### Study cohort

A total of 368 patient proxy pairs were screened and 204 of those were recruited between 25 th of February 2019 and 27 th of July 2020. 102 (50%) patients were postoperatively admitted to the ICU (Figure S1). Patients were 41% females, had a median age of 69 [interquartile range (IQR) 59–77] years and were mainly admitted for visceral or cardiac surgery. The median hospital length of stay was 13.0 [IQR 8.0–21.0] days, with a median ICU length of stay of 3.0 [IQR 1.0–5.0] days. Patients admitted to the ICU postoperatively were significantly older, had more comorbidities requiring medication, were mostly cardiac surgical patients, and presented worse pre-operative PROMs compared to those admitted to the normal ward postoperatively (Table 1). Their proxies had a median age of 64 [IQR 53–71] years, were 68% females and were mostly spouses with a common household, therefore seeing each other daily. Thirty-eight postoperative interviews were conducted in the ICU (Table 2).

**Table 1:** Baseline characteristics patients

Variable	All	NW	ICU	P value
n	204	102	102	
Age (years)	69 [59–77]	67 [58–73]	72 [64–79]	0.001
Sex, female	83 [40.7]	42 [41.2]	41 [40.2]	0.89
Hospital length of stay (days)	13 [8–21]	15 [9–23]	11 [7–20]	0.15
ICU length of stay (days)	N/A	N/A	3 [1–5]	
Pre-existing conditions (count)	3 (2–6)	2 (1–4)	6 (3–9)	< 0.001
Previous surgeries (count)	2 (1–3)	2 (1–3)	2 (1–3)	0.71
Pre-existing medication (count)	3 (1–7)	2 (0–4)	6 (3–9)	< 0.001
Surgical discipline				
Orthopaedic/trauma	10 (4.9)	10 (9.8)	0 (0)	< 0.001
Visceral surgery	89 (43.6)	57 (55.9)	32 (31.4)	
Cardiac surgery	63 (30.9)	0 (0)	63 (61.8)	
Thoracic/vascular surgery	21 (10.3)	19 (18.6)	2 (2.0)	
Neurosurgery	13 (6.4)	11 (10.8)	2 (2.0)	
Gynaecology	8 (3.9)	5 (4.9)	3 (2.9)	
Patient-reported Outcome Measures				
WHODAS 2.0	12.5 [4.2–26.0]	8.3 [2.1–25.0]	16.7 [8.3–29.2]	0.001
EQ-5D Index Value	0.89 [0.64–1.00]	0.94 [0.60–1.00]	0.86 [0.69–0.96]	0.028
Barthel-Index	100 [100–100]	100 [100–100]	100 [95–100]	0.27
SF-36 Physical Component Score	41.7 [32.2–50.6]	46.9 [35.2–53.8]	40.0 [31.2–47.8]	< 0.001
SF-36 Mental Component Score	52.3 [45.4–58.0]	51.7 [44.4–56.8]	53.6 [46.2–59.2]	0.09

Continuous variables are shown as median [interquartile range], categorical as number (percentage). The significance level between patients admitted to the ward and ICU was determined with the Mann–Whitney U test for continuous variables and the Chi-Square test for categorical variables

*ICU* intensive care unit; *NW* normal ward

**Table 2:** Baseline characteristics of proxies

		All	NW	ICU	p-value
		N = 204	N = 102	N = 102	
Age (years)		64 [53–71]^a^	63 [52–70]	66 [54–73]	0.184
Sex	Female	139 (68.0)	73 (71.3)	76 (64.7)	0.315
Relationship status	Married	120 (58.8)	65 (63.7)	55 (53.9)	0.028
	Partnership	17(8.3)	7 (6.9)	10 (9.8)	
	Parents	8 (3.9)	4 (3.9)	4 (3.9)	
	Sibling	17 (8.3)	13 (12.7)	4 (3.9)	
	Child	37 (18.1)	12 (11.8)	25 (24.5)	
	Others	5 (2.5)	1 (1.0)	4 (3.9)	
	If married, duration (years)	40 (22–48)	33 (18–46)	45 (32–50)	0.007
	If partnered, duration (years)	18 (10–21)	20 (10–21)	15 (10–21)	0.837
Shared household	Yes	143 (70.1)	72 (70.6)	71 (69.9)	0.785
	No	14 (6.9)	8 (7.8)	6 (5.9)	
	Not any more	47 (23.0)	22 (21.6)	25 (24.5)	
Visits	Daily	149 (73.0)	76 (74.55)	73 (71.6)	0.627
	At least 1 per week	23 (11.3)	9 (8.8)	14 (13.7)	
	At least 1 per month	17 (8.3)	10 (9.8)	7 (6.9)	
	Less than 1 per month	15 (7.4)	7 (6.9)	8 (7.8)	
Calls^b^	Daily	85 (46.4)	45 (45.5)	40 (47.6)	0.936
	At least 1 per week	57 (31.1)	30 (30.3)	27 (32.1)	
	At least 1 per month	17 (9.3)	10 (10.1)	7 (8.3)	
	Less than 1 per month	24 (13.1)	14 (14.1)	10 (11.9)	
Text message^c^	Daily	76 (41.8)	41 (40.6)	35 (43.2)	0.174
	At least 1 per week	32 (17.6)	20 (19.8)	12 (14.8)	
	At least 1 per month	14 (7.7)	11 (10.9)	3 (3.7)	
	Less than 1 per month	60 (33.0)	29 (28.7)	31 (38.3)	
	ICU	38 (18.6)	0 (0)	38 (37.3)	

Continuous variables are shown as median [interquartile range]. Categorical variables are shown as count (percentage). The significance level between patients admitted to the ward and ICU was determined with the Mann–Whitney U test for continuous variables and the Chi-Square test for categorical variables

*ICU* Intensive Care Unit; *NW* Normal Ward

^a^4 missing values

^b^21 missing values

^c^22 missing

### Study short-form health survey 36 (SF-36)

**Fig. 1:**
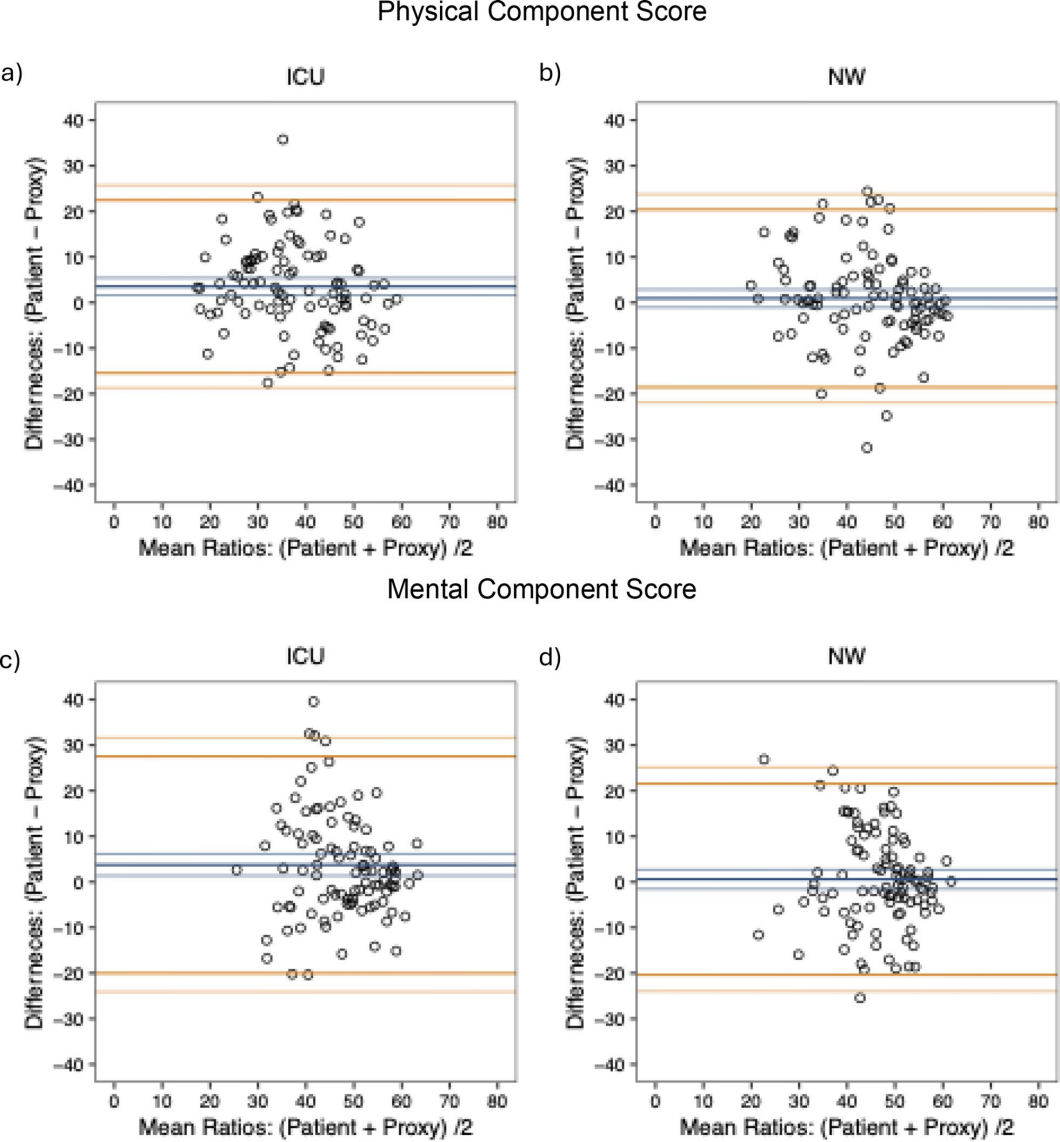
Reliability for regular ward and ICU in Bland–Altman plots for the SF-36. Displayed are the mean value of the response from patients and proxies on the x-axis and the difference between their answers on the y-axis. The blue lines indicate the mean difference and the corresponding 95% CI. The yellow lines indicate the upper and lower 95% limits of agreement with the respective 95% CI. Panel **a** shows the Physical Component Score for the ICU cohort and **b** for the normal ward cohort while **c** shows the Mental Component Score for the ICU cohort and **d** for the normal ward cohort. The SF-36 PCS (normal ward – mean difference [95% limits of agreement]: 0.9 [− 18.6 to 20.4]; ICU – mean difference [95% limits of agreement]: 3.5 [− 15.4 to 22.5]) shows insufficient reliability and a significant bias in the ICU patients (normal ward – mean difference [95% CI]: 0.9 [− 1.0 to 2.9]; ICU – mean difference [95%CI]: 3.5 [1.6–5.4]). The SF-36 MCS (normal ward – mean difference [95% limits of agreement]: 0.6 [− 20.4 to 21.5]; ICU – mean difference [95% limits of agreement]: 3.8 [− 20.0 to 27.5]) shows insufficient reliability and a significant bias in the ICU patients (normal ward – mean difference [95% CI]: 0.6 [− 1.5 to 2.7]; ICU – mean difference [95%CI]: 3.8 [1.4–6.2]). *ICU* intensive care unit; *NW* normal ward

The Bland–Altman plots showed 95% limits of agreement for the PCS and MCS widely outside the MCID of 5 for the PCS and 5.5 for the MCS [28] for both the ICU (Fig. 1a, c) and normal ward (Fig. 1b, d) patients, indicating insufficient reliability. This was corroborated by the dichotomous analysis, which showed a high proportion of pairs that had scoring difference outside the MCID. Normal ward and ICU patients displayed similar proportions in this analysis (Table 3). While for normal ward patients, no bias was present, a significant bias towards a worse evaluation by the proxies for the PCS and MCS in ICU patients was observed (Fig. 1a–d). Sensitivity analysis without imputation showed similar results (Supplementary File 1: Figure S1 and Table S1).

**Table 3:** Patient Proxy Pairs with a Difference outside the MCID

Variable	Normal ward (n = 102)	ICU (n = 102)	p-value
SF-36 physical component scale	47 (46.1%)	58 (56.9%)	0.120
SF-36 mental component scale	59 (57.8%)	62 (60.8%)	0.700
WHODAS 2.0	58 (56.9%)	78 (76.5%)	0.003
EQ-5D-5L	40 (39.2%)	46 (45.1)	0.400
Barthel-Index	22 (21.6%)	21 (20.6%)	0.900

Shown is the number and percentage of patient-proxy pairs displaying a difference between the scores larger than the MCID. Normal ward and ICU patients were compared with the Chi-squared test

### WHO disability assessment schedule 2.0

**Fig. 2:**
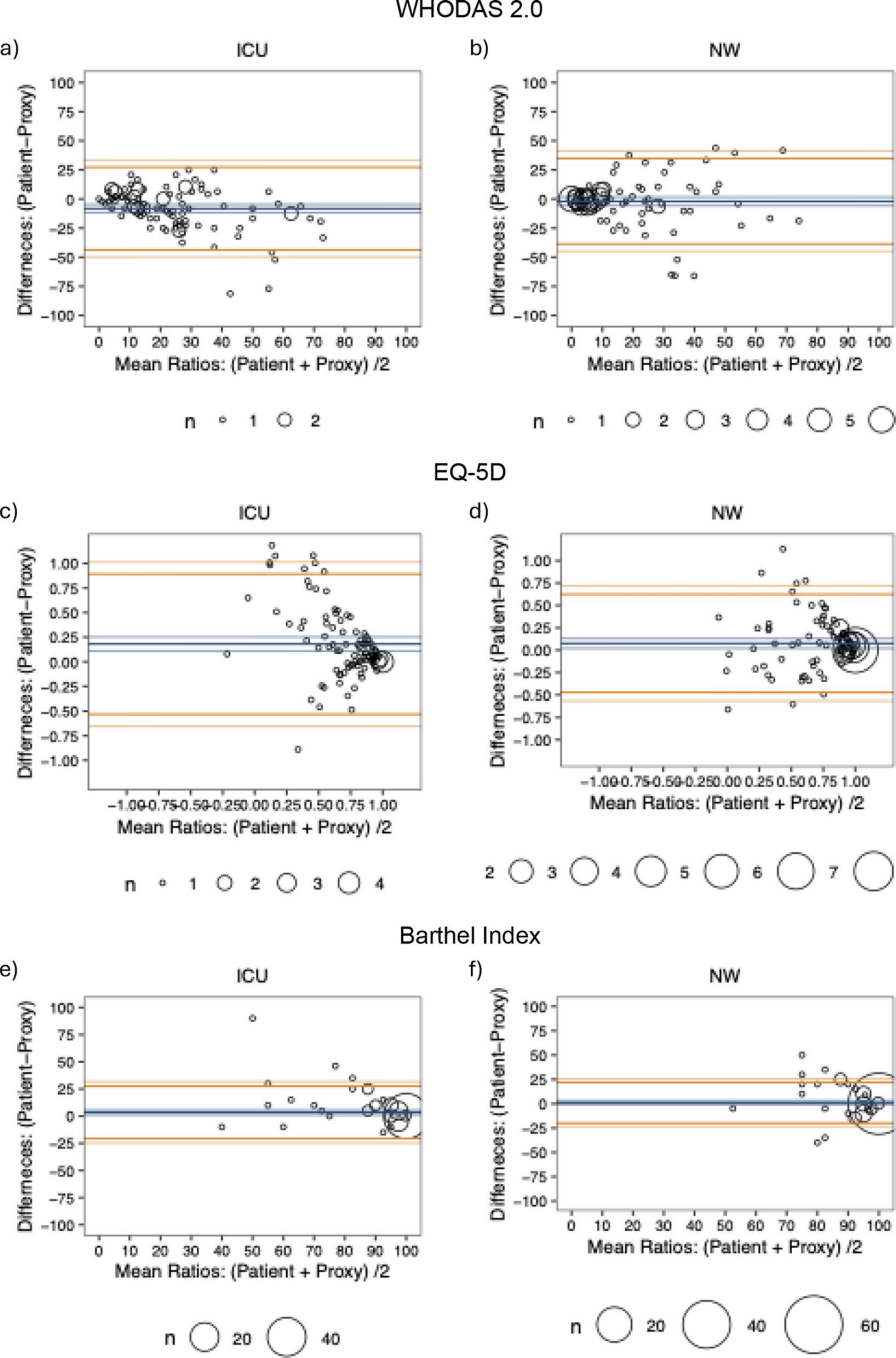
Reliability for regular ward and ICU in Bland–Altman plots for the WHODAS 2.0, Barthel-Index and IADL. Displayed are the mean value of the response from patients and proxies on the x-axis and the difference between their answers on the y-axis. The blue lines indicate the mean difference and the corresponding 95% CI. The yellow lines indicate the upper and lower 95% limits of agreement with the respective 95% CI. Panel **a** shows the WHODAS 2.0 for the ICU cohort and **b** for the normal ward cohort, **c** shows the EQ-5D for the ICU cohort and **d** for the normal ward cohort while **e** shows the Barthel Index for the ICU cohort and **f** for the normal ward cohort. The WHODAS 2.0 (normal ward – mean difference [95% limits of agreement]: − 2.0 [− 38.7 to 34.8]; ICU – mean difference [95% limits of agreement]: − 8.2 [− 43.7 to 27.3]) shows insufficient reliability with a significant bias in the ICU patients (normal ward – mean difference [95% CI]: − 2.0 [− 5.6 to 1.7]; ICU – mean difference [95%CI]: − 8.2 [− 11.8 to − 4.6]). The EQ-5D (normal ward – mean difference [95% limits of agreement]: 0.1 [− 0.5 to 0.6]; ICU – mean difference [95% limits of agreement]: 0.2 [− 0.5 to 0.9]) shows insufficient reliability without a bias (normal ward – mean difference [95% CI]: 0.1 [0.0–0.1]; ICU – mean difference [95%CI]: 0.2 [0.1 to − 0.3]). The Barthel (normal ward – mean difference [95% limits of agreement]: 1.1 [− 19.9 to 22.0]; ICU – mean difference [95% limits of agreement]: 3.5 [− 20.6 to 27.5]) shows insufficient reliability with a significant bias in ICU patients (normal ward – mean difference [95% CI]: 1.1 [− 1.0 to 3.2]; ICU – mean difference [95%CI]: 3.5 [1.0 to 5.9]). *ICU* intensive care unit; *NW* normal ward

Similar to the SF-36, the Bland–Altman plots demonstrated 95% limits of agreement for the WHODAS 2.0 widely outside the MCID of 5 [26] for both the ICU (Fig. 2a) and normal ward (Fig. 2b) patients, indicating insufficient reliability. Moreover, a significant bias towards a worse evaluation by the proxies was evident in ICU patients, which could not be identified in normal ward patients (Fig. 2a, b). This was again corroborated by the dichotomous analysis, which showed a more significant proportion of ICU patients outside the MCID than normal ward patients (Table 3). Sensitivity analysis without imputation showed similar results (Supplementary File 1 Figure S2 and Table S1).

### EQ-5D

The Bland–Altman presented 95% limits of agreement again outside the MCID of 0.18 [29] in the ICU (Fig. 2c) and normal ward (Fig. 2d) patients, indicating insufficient reliability also for EQ-5D. Additionally, a significant bias towards a worse evaluation by the proxies was present in both cohorts. The dichotomous analysis also pointed towards insufficient reliability, although there was no significant difference between ICU and normal ward patients (Table 3). Sensitivity analysis without imputation showed similar results (Supplementary File 1: Figure S3 and Table S1).

### Barthel Index

The Bland–Altman plots showed that the 95% limits of agreement extend beyond the MCID of 9.8 [30] in ICU (Fig. 2e) and normal ward (Fig. 2f) patients, indicating insufficient reliability for the Barthel Index. Furthermore, a significant bias towards a worse evaluation by the proxies could be observed in ICU patients. These results were underlined by the dichotomous analysis, with a relevant proportion of ICU and normal ward patients displaying a difference outside the MCID (Table 3). Sensitivity analysis without imputation showed similar results (Supplementary File 1 Figure S2 and Table S1).

### Instrumental activities of daily living

Agreement for IADL was “fair” when evaluated with unweighted values and improved to “moderate” when evaluated with weighted kappa values (Table S2). The sensitivity analysis confirmed those findings (Supplementary File 1 Table S3).

### Subgroup analyses

We conducted a subgroup analysis considering the frequency of contact between the patient and proxy, along with shared household status for both parties, as indicators of a close relationship. Neither variable demonstrated a significant difference in the proportion of assessments showing a difference below the MCID (Supplementary File 1 Tables S4 and S5).

## Discussion

This is the first study investigating the reliability of the pre-admission PROM assessment via proxies in a collective of surgical patients focusing on post-operative ICU patients who reported their PROMs prior to surgery and the respective ICU admission. We were able to demonstrate that the reliability of the assessment of the SF-36, WHODAS 2.0, IADL, EQ5D-5L, and Barthel Index by a proxy was insufficient. Furthermore, a significant bias was present in ICU patients with a worse evaluation by the proxies compared to the normal ward patients, indicating that the ICU admission or stay appeared to be a substantial confounder in the assessment via proxies.

Proxies, such as children, spouses, and friends, experience severe sleep disorders, anxiety, and fatigue during the ICU treatment of their loved ones, which may affect their judgment [31, 32]. These factors could cause recall bias and consequently influence the assessment of pre-admission status via PROMs, explaining the insufficient reliability and observed bias. Our findings and this assumption align with the investigation by Rogers et al., who also evaluated the reliability of proxy assessments of the SF-36 in ICU patients. They demonstrated that reliability is compromised, showing a significant bias towards a poorer evaluation by proxies, which is consistent with our results. However, they collected scores for assessing admission status at ICU discharge and six months later, revealing improved reliability 6 months after discharge, thus reinforcing the argument for the significant impact of the ICU setting on proxy assessments [12]. At the time of ICU admission, when the patient's outcome is uncertain, this bias may be even more pronounced. Furthermore, it remains unclear whether patients and proxies with certain characteristics, such as a prolonged relationship or cohabitation, exhibit better reliability. This question should be investigated in future studies.

The impact of the setting was further supported by an evaluation of the reliability of proxy assessments in 96 elective cardiac surgery patients, conducted prior to surgery, at hospital discharge, and 6 months after discharge. The lowest level of agreement was found at hospital discharge [33]. These studies confirm our observation of bias within the ICU cohort that was absent in the normal ward cohort, suggesting that this bias emerged from the influence of the proxies'current situation.

Since no objective measures were performed, it remains unclear whether patients’ or proxies’ responses were more accurate. Multiple factors that could influence patients’ self-reports exist in the ICU context. Response shift, or the change in self-reported quality of life concerning the time point and circumstances, was a confounding factor in previous trials that retrospectively assessed pre-admission status [34]. This shift was observed in other groups, such as severely mobility-disabled individuals [35]. On the other hand, PTSD and depression are common challenges following critical illness [36, 37]. Furthermore, lower PROM ratings and more chronic conditions may be evident in the ICU cohort, which could contribute to the observed bias between patients and proxies in this group. These factors could be confounders skewing the patients’ self-evaluation, either positively or negatively. This was highlighted in the work by Dinglas et al., who assessed the differences between patient and proxy evaluations conducted at hospital discharge regarding pre-admission baseline status. They identified a non-linear bias in their Bland–Altman analysis, showing that proxies tended to diminish patients’ responses; specifically, when high patient scores were reported, lower proxy ratings followed, and conversely, when lower patient scores were noted, higher proxy ratings appeared [34]. This further indicates that bias is not uniform and depends on variables such as the instrument used, severity of illness, outcomes of critical illness, and prior functional status. Our results reflect this, as the reliability of the IADL, which employs a clearly defined scale, tends to exhibit superior reliability compared to scores utilizing a more subjective scale.

Health-related quality-of-life instruments are subjective, making them more challenging to report by a proxy. These factors are illustrated in the study by Elliott et al., who found better agreement on more objective measurements, such as physical function, and worse agreement for subjective areas, such as vitality [33]. This emphasizes the need for further research to identify and implement outcome measures that are resistant to confounding by proxy assessment for critically ill patients.

The study by Hofhuis et al. is the only one that examined the reliability of a proxy assessment of patients' health-related quality of life at ICU admission, similar to ours. They utilised the SF-36 to evaluate health-related quality of life. Their study demonstrated a significant difference between patients and proxies across the various SF-36 domains, except for physical and emotional roles [13]. This aligns with our findings, which indicate poor reliability. However, the applicability of their study to real-life scenarios is limited, as their patients could complete the questionnaires themselves upon ICU admission. Nonetheless, Scales et al. produced similar results in a cohort of ARDS patients when the proxy was interviewed at admission and the patient later [38]. Consistent with the SF-36 results, the EQ-5D also performs poorly when obtained via proxies [34]. No studies are available on the other assessment tools that were explored. A strength of the conducted study is its multicentric design and the assessment of patients and proxies at different time points—patients before surgery and ICU admission, as well as proxies after surgery and ICU admission. This design reflects a real-world scenario where proxies are interviewed after patients have been admitted to the ICU. This approach allows for assessing the ICU as a confounding factor for the proxy response while excluding it as a confounder for the patient’s response, as might occur if the patient were evaluated in the ICU. Another strength is the heterogeneity within the cohort, which facilitates generalisation to a broad population of surgical ICU patients.

The results were limited by the subjective nature of the assessment performed, as they did not permit identification of whether the patient's or proxy's response was closer to the objective performance status. Nevertheless, the patient’s ratings serve as the gold standard, independent of objective assessments, for patient-reported outcome measures like quality of life. Furthermore, accurate functional measurements at ICU admission are often unattainable due to ventilation or sedation. A second limitation was that our cohort included solely elective surgical patients who were able to complete the questionnaires preoperatively. It is unclear whether medical ICU patients, emergency admissions, and their proxies are similarly biased. Additionally, emergency admissions likely introduced a greater bias, as the aforementioned confounding factors may increase due to the unexpected nature of the illness and its impact on proxies. Both these aspects should be investigated in future trials. Cultural and socioeconomic factors were not examined separately and could be potential confounders. These factors might also result in response bias, as external expectations may skew the proxy responses. To date, there is no established core outcome set for assessing patients prior to surgeries associated with a high likelihood of ICU admission, nor for the pre-admission ICU status of surgical patients. The questionnaires used represent a sample of various core outcome sets applied in different critical care research topics. Furthermore, part of the recruitment occurred during the SARS-CoV-2 pandemic, and its impact on critical care, as a central focus of mainstream media highlighting high mortality rates, cannot be evaluated.

In conclusion, the proxy assessment of pre-admission PROMs demonstrated insufficient reliability and exhibited significant bias in ICU patients when using commonly utilised questionnaires (SF-36, WHODAS 2.0, ED-5L, and Barthel Index). Therefore, it should be applied cautiously and not interchangeably in analyses with patient reports. Future research into objective or robust subjective measures is urgently needed to adequately inform treatment decisions, study inclusion, and the development of an adequate ICU pre-admission assessment.

## Supplementary Information

Supplementary Material 1.

## Data Availability

The datasets generated and/or analysed during the current study are not publicly available due to German data protection laws but are available from the corresponding author in anonymised form on reasonable scientific request.

## Abbreviations

ICU: Intensive care unit
PROM: Patient-reported outcome measure
MCID: Minimal clinically important difference
IADL: Instrumental activities of daily living
WHODAS 2.0: World Health Organization Disability Assessment Schedule
SF-36: Medical Outcome Study Short-Form Health Survey 36
MCS: Mental Component Score
PCS: Physical Component Score
IQR: Interquartile range
SD: Standard deviation

## References

[1] Needham DM, Davidson J, Cohen H, Hopkins RO, Weinert C, Wunsch H, et al. Improving long-term outcomes after discharge from intensive care unit: report from a stakeholders’ conference. Crit Care Med. 2012;40(2):502–9. 21946660 10.1097/CCM.0b013e318232da75

[2] Bagshaw SM, Webb SA, Delaney A, George C, Pilcher D, Hart GK, et al. Very old patients admitted to intensive care in Australia and New Zealand: a multi-centre cohort analysis. Crit Care. 2009;13(2):R45. 19335921 10.1186/cc7768 PMC2689489

[3] Wehler M, Strauss R, Bost A, Geise A, Mueller A, Martus P, et al. Pre-admission functional status and outcome in medical intensive care. Crit Care. 2000;4(Suppl 1):P225.

[4] Krinsley JS, Wasser T, Kang G, Bagshaw SM. Pre-admission functional status impacts the performance of the APACHE IV model of mortality prediction in critically ill patients. Crit Care. 2017;21(1):110. 28506290 10.1186/s13054-017-1688-z PMC5433010

[5] Bagshaw SM, Stelfox HT, McDermid RC, Rolfson DB, Tsuyuki RT, Baig N, et al. Association between frailty and short- and long-term outcomes among critically ill patients: a multicentre prospective cohort study. CMAJ. 2014;186(2):E95-102. 24277703 10.1503/cmaj.130639 PMC3903764

[6] Sprung CL, Joynt GM, Christian MD, Truog RD, Rello J, Nates JL. Adult ICU triage during the coronavirus disease 2019 pandemic: who will live and who will die? recommendations to improve survival. Crit Care Med. 2020;48(8):1196–202. 32697491 10.1097/CCM.0000000000004410 PMC7217126

[7] Paton M, Lane R, Paul E, Linke N, Shehabi Y, Hodgson CL. Correlation of patient-reported outcome measures to performance-based function in critical care survivors: predictable. Aust Crit Care. 2023;36(4):485–91. 35810078 10.1016/j.aucc.2022.05.006

[8] Cohen RI, Eichorn A, Silver A. Admission decisions to a medical intensive care unit are based on functional status rather than severity of illness. A single center experience. Minerva Anestesiol. 2012;78(11):1226–33. 22699698

[9] Li M, Harris I, Lu ZK. Differences in proxy-reported and patient-reported outcomes: assessing health and functional status among medicare beneficiaries. BMC Med Res Methodol. 2015;15:62. 26264727 10.1186/s12874-015-0053-7 PMC4534114

[10] Liang Y, Che T, Zhang H, Shang L, Zhang Y, Xu Y, et al. Assessing the proxy response bias of EQ-5D-3 L in general population: a study based on a large-scale representative household health survey using propensity score matching. Health Qual Life Outcomes. 2020;18(1):75. 32188480 10.1186/s12955-020-01325-z PMC7079393

[11] Hernandez JD, Spir MA, Payares K, Posada AM, Salinas FA, Garcia HI, et al. Assessment by proxy of the SF-36 and WHO-DAS 2.0. A systematic review. J Rehabil Med. 2023;55:jrm4493. 37389563 10.2340/jrm.v55.4493 PMC10337773

[12] Rogers J, Ridley S, Chrispin P, Scotton H, Lloyd D. Reliability of the next of kins’ estimates of critically ill patients’ quality of life. Anaesthesia. 1997;52(12):1137–43. 9485965 10.1111/j.1365-2044.1997.240-az0374.x

[13] Hofhuis J, Hautvast JLA, Schrijvers AJP, Bakker J. Quality of life on admission to the intensive care: can we query the relatives? Intensive Care Med. 2003;29(6):974–9. 12734653 10.1007/s00134-003-1763-6

[14] Davies TW, van Gassel RJJ, van de Poll M, Gunst J, Casaer MP, Christopher KB, et al. Core outcome measures for clinical effectiveness trials of nutritional and metabolic interventions in critical illness: an international modified Delphi consensus study evaluation (CONCISE). Crit Care. 2022;26(1):240. 35933433 10.1186/s13054-022-04113-x PMC9357332

[15] Needham DM, Sepulveda KA, Dinglas VD, Chessare CM, Friedman LA, Bingham CO, et al. Core outcome measures for clinical research in acute respiratory failure survivors. An International Modified Delphi Consensus Study. Am J Resp Crit Care. 2017;196(9):1122–30. 10.1164/rccm.201702-0372OC PMC5694837 28537429

[16] Ware JE Jr, Kosinski M, Bayliss MS, McHorney CA, Rogers WH, Raczek A. Comparison of methods for the scoring and statistical analysis of SF-36 health profile and summary measures: summary of results from the Medical Outcomes Study. Med Care. 1995;33(4 Suppl):AS264–79. 7723455

[17] Ware JE Jr, Sherbourne CD. The MOS 36-item short-form health survey (SF-36). I. Conceptual framework and item selection. Med Care. 1992;30(6):473–83. 1593914

[18] Ustun TB, Chatterji S, Kostanjsek N, Rehm J, Kennedy C, Epping-Jordan J, et al. Developing the World Health Organization Disability Assessment Schedule 2.0. Bull World Health Organ. 2010;88(11):815–23. 21076562 10.2471/BLT.09.067231 PMC2971503

[19] Herdman M, Gudex C, Lloyd A, Janssen M, Kind P, Parkin D, et al. Development and preliminary testing of the new five-level version of EQ-5D (EQ-5D-5L). Qual Life Res. 2011;20(10):1727–36. 21479777 10.1007/s11136-011-9903-x PMC3220807

[20] Lawton MP, Brody EM. Assessment of older people: self-maintaining and instrumental activities of daily living. Gerontologist. 1969;9(3):179–86. 5349366

[21] Nasreddine ZS, Phillips NA, Bédirian V, Charbonneau S, Whitehead V, Collin I, et al. The Montreal Cognitive Assessment, MoCA: a brief screening tool for mild cognitive impairment. J Am Geriatr Soc. 2005;53(4):695–9. 15817019 10.1111/j.1532-5415.2005.53221.x

[22] Mahoney FI, Barthel DW. Functional evaluation: the Barthel Index. Md State Med J. 1965;14:61–5. 14258950

[23] Bland JM. How can I decide the sample size for a study of agreement between two methods of measurement? 2004; updated 12.01.2004. Available from: https://www-users.york.ac.uk/~mb55/meas/sizemeth.htm.

[24] Hodgson CL, Udy AA, Bailey M, Barrett J, Bellomo R, Bucknall T, et al. The impact of disability in survivors of critical illness. Intensive Care Med. 2017;43(7):992–1001. 28534110 10.1007/s00134-017-4830-0

[25] Üstün TB KN, Chatterji S, Rehm J. Measuring health and disability manual for WHO Disability Assessment Schedule WHODAS 2.0: World Health Organization; 2010.

[26] Shulman MA, Kasza J, Myles PS. Defining the minimal clinically important difference and patient-acceptable symptom state score for disability assessment in surgical patients. Anesthesiology. 2020;132(6):1362–70. 32167984 10.1097/ALN.0000000000003240

[27] Ludwig K, Graf von der Schulenburg JM, Greiner W. German value set for the EQ-5D-5L. Pharmacoeconomics. 2018;36(6):663–74. 29460066 10.1007/s40273-018-0615-8 PMC5954069

[28] Haines KJ, Berney S, Warrillow S, Denehy L. Long-term recovery following critical illness in an Australian cohort. J Intensive Care. 2018;6:8. 29445502 10.1186/s40560-018-0276-x PMC5800039

[29] (ISPOR) TPSfHEaOR. Minimal clinically important difference in EQ-5D: we can calculate it—but does that mean we should? [PowerPoint]. Webpage: ISPOR; 2017 [PowerPoint]. Available from: https://www.ispor.org/docs/default-source/presentations/1066.pdf.

[30] Unnanuntana A, Jarusriwanna A, Nepal S. Validity and responsiveness of Barthel index for measuring functional recovery after hemiarthroplasty for femoral neck fracture. Arch Orthop Trauma Surg. 2018;138(12):1671–7. 30094561 10.1007/s00402-018-3020-z

[31] Williamson AM, Feyer AM. Moderate sleep deprivation produces impairments in cognitive and motor performance equivalent to legally prescribed levels of alcohol intoxication. Occup Environ Med. 2000;57(10):649–55. 10984335 10.1136/oem.57.10.649 PMC1739867

[32] Day A, Haj-Bakri S, Lubchansky S, Mehta S. Sleep, anxiety and fatigue in family members of patients admitted to the intensive care unit: a questionnaire study. Crit Care. 2013;17(3):R91. 23705988 10.1186/cc12736 PMC3706908

[33] Elliott D, Lazarus R, Leeder SR. Proxy respondents reliably assessed the quality of life of elective cardiac surgery patients. J Clin Epidemiol. 2006;59(2):153–9. 16426950 10.1016/j.jclinepi.2005.06.010

[34] Dinglas VD, Gifford JM, Husain N, Colantuoni E, Needham DM. Quality of life before intensive care using EQ-5D: patient versus proxy responses. Crit Care Med. 2013;41(1):9–14. 23232287 10.1097/CCM.0b013e318265f340 PMC3531666

[35] Stensman R. Severely mobility-disabled people assess the quality of their lives. Scand J Rehabil Med. 1985;17(2):87–99. 3161178

[36] Patel MB, Jackson JC, Morandi A, Girard TD, Hughes CG, Thompson JL, et al. Incidence and risk factors for intensive care unit-related post-traumatic stress disorder in veterans and civilians. Am J Respir Crit Care Med. 2016;193(12):1373–81. 26735627 10.1164/rccm.201506-1158OC PMC4910886

[37] Rabiee A, Nikayin S, Hashem MD, Huang M, Dinglas VD, Bienvenu OJ, et al. Depressive symptoms after critical illness: a systematic review and meta-analysis. Crit Care Med. 2016;44(9):1744–53. 27153046 10.1097/CCM.0000000000001811 PMC7418220

[38] Scales DC, Tansey CM, Matte A, Herridge MS. Difference in reported pre-morbid health-related quality of life between ARDS survivors and their substitute decision makers. Intensive Care Med. 2006;32(11):1826–31. 16957904 10.1007/s00134-006-0333-0

