# Supplementary material for: Reliability of pre-admission patient-reported outcome measures postoperatively assessed via proxies: a prospective, multicenter observational study
Source: Crit Care. 2025 May 19;29:200. doi: 10.1186/s13054-025-05431-6 (PMC12090533; doi:10.1186/s13054-025-05431-6)
Supplement: Supplementary file 1 — Supplementary Material 1. [file 13054_2025_5431_MOESM1_ESM.pdf]

Additional File 1

Reliability of pre-admission Patient-reported Outcome Measures postoperatively assessed via Proxies: a prospective, multicenter observational study

Authors

Julius J. Grunow[1] (0000-0003-4164-0138), Leonie Hartmann[1], Bernhard Ulm[2,3], Mathilde Maechler[1], Manfred Blobner[2,3], Katharina Seidenspinner[4], Lukas Schoennagel[1], Steffen Weber-Carstens[1], Kristina Fuest[3], Tobias Wollersheim[1,+], Stefan J Schaller[1,2,5,+] (0000-0002-6683-9584)

+ These authors contributed equally.

Affiliations

1. Charité – Universitätsmedizin Berlin, corporate member of Freie Universität Berlin and Humboldt Universität zu Berlin, Department of Anaesthesiology and Intensive Care Medicine (CCM/CVK)
2. Technical University of Munich, School of Medicine and Health, Klinikum rechts der Isar, Department of Anaesthesiology and Intensive Care Medicine
3. University of Ulm, Faculty of Medicine, Department of Anesthesiology and Intensive Care Medicine, Ulm, Germany
4. Department of Cardiology, Vivantes Klinikum im Friedrichshain, Berlin, Germany
5. Medical University of Vienna, Department of Anaesthesia, Intensive Care Medicine and Pain Medicine, Division of General Anaesthesia and Intensive Care Medicine, Vienna, Austria

Corresponding Author

Univ.-Prof. Dr. med. Stefan J. Schaller

Charité – Universitätsmedizin Berlin

Department of Anaesthesiology and Intensive Care Medicine (CCM/CVK)

Chariteplatz 1

10117 Berlin

Germany

stefan.schaller@charite.de

Table of Content

TABLE OF CONTENT 2

SUPPLEMENTARY TABLES 3

Table S1. Patient Proxy Pairs with a Difference outside the MCID 3

Table S2. Cohen's Kappa for IADL 4

Table S3. Cohen's Kappa for IADL without imputed values 5

Table S4. Patient Proxy Pairs with a Difference outside the MCID according to Household Status 6

Table S5. Patient Proxy Pairs with a Difference outside the MCID according to Frequency of Visits 7

SUPPLEMENTARY FIGURES 8

Figure S1. Study flow chart 8

Figure S2. Reliability for regular ward and ICU in Bland-Altmann plots for the SF-36 9

Figure S3. Reliability for regular ward and ICU in Bland-Altmann plots for WHODAS 2.0, Barthel-Index and IADL. 10

Supplementary Tables

Table S1. Patient Proxy Pairs with a Difference outside the MCID

Variable	Normal Ward (n = 102)	ICU (n = 102)	p-value
SF-36 Physical component scale	41 (46%)	53 (58%)	0.14
SF-36 Mental component scale	51 (57%)	59 (64%)	0.37
WHODAS 2.0	57 (56%)	78 (77%)	0.003
EQ-5D-5L	38 (39%)	45 (46%)	0.39
Barthel-Index	19 (20%)	20 (20%)	1.0

Shown is the number and percentage of patient-proxy pairs displaying a difference between the scores larger than the MCID. Normal ward and ICU patients were compared with the Chi-squared test.
MCID, minimal clinically important difference

Table S2. Cohen's Kappa for IADL

Variable	All	Normal Ward	ICU	p-value
IADL unweighted#	0.26 [0.18 – 0.35]	0.25 [0.12 – 0.39]	0.27 [0.16 – 0.37]	0.93
IADL weighted#	0.43 [0.33 – 0.52]	0.41 [0.26 – 0.57]	0.43 [0.32 – 0.55]	0.89

Cohens's Kappa weighted and unweighted with 95% confidence interval for Instrumental Activities of Daily Living

Table S3. Cohen’s Kappa for IADL without imputed values

Variable	All	Normal Ward	ICU	p-value
IADL unweighted#	0.29 [0.20 – 0.38]	0.27 [0.15 – 0.39]	0.30 [0.16 – 0.45]	0.84
IADL weighted#	0.48 [0.37 – 0.59]	0.48 [0.34 – 0.62]	0.47 [0.30 – 0.65]	0.98

Cohens’s Kappa weighted and unweighted with 95% confidence interval for Instrumental Activities of Daily Living

Table S4. Patient Proxy Pairs with a Difference outside the MCID according to Household Status

Shared Household		Yes	No	Not Anymore	p-value
Barthel Index	Inside MCID	110 (80.9%)	10 (71.4%)	35 (79.5%)	0.701
	Outside MCID	26 (19.1%)	4 (28.6%)	9 (20.5%)	
EQ-5D-5L	Inside MCID	84 (60.0%)	8 (61.5%)	22 (50.0%)	0.484
	Outside MCID	56 (40.0%)	5 (38.5%)	22 (50.0%	
WHODAS 2.0	Inside MCID	47 (33.1)	6 (42.9%)	14 (30.4%)	0.688
	Outside MCID	95 (66.9%)	8 (57.1%)	32 (69.6%)	
SF-36 Physical component score	Inside MCID	60 (46.9%)	8 (66.7%)	19 (46.3%)	0.410
	Outside MCID	68 (53.1%)	4 (33.3%)	22 (53.7%)	
SF-36 Mental component score	Inside MCID	51 (39.8%)	4 (33.3%)	16 (39.0%)	0.907
	Outside MCID	77 (60.2%)	8 (66.7%)	25 (61.0%)	

Shown is the number and percentage of patient-proxy pairs displaying a difference between the scores larger or smaller than the MCID. Normal ward and ICU patients were compared with the Chi-squared test.

Table S5. Patient Proxy Pairs with a Difference outside the MCID according to Frequency of Visits

**Shared Household**		**Daily**	**Less than Daily**	**p-value**
Barthel Index	Inside MCID	115 (81.0%)	40 (76.9)	0.548
	Outside MCID	27 (19.0%)	12 (23.1%)	
EQ-5D-5L	Inside MCID	85 (58.6%)	29 (55.8%)	0.745
	Outside MCID	60 (41.4%)	23 (44.2%)	
WHODAS 2.0	Inside MCID	50 (33.8%)	17 (31.5%)	0.866
	Outside MCID	98 (66.2%)	37 (68.5%)	
SF-36 Physical component score	Inside MCID	63 (47.4%)	24 (50.0%)	0.866
	Outside MCID	70 (52.6%)	24 (50.0%)	
SF-36 Mental component score	Inside MCID	50 (37.6%)	21 (43.8%)	0.493
	Outside MCID	83 (62.4%)	27 (56.3%)	

Shown is the number and percentage of patient-proxy pairs displaying a difference between the scores larger or smaller than the MCID. Normal ward and ICU patients were compared with the Chi-squared test.

Supplementary Figures

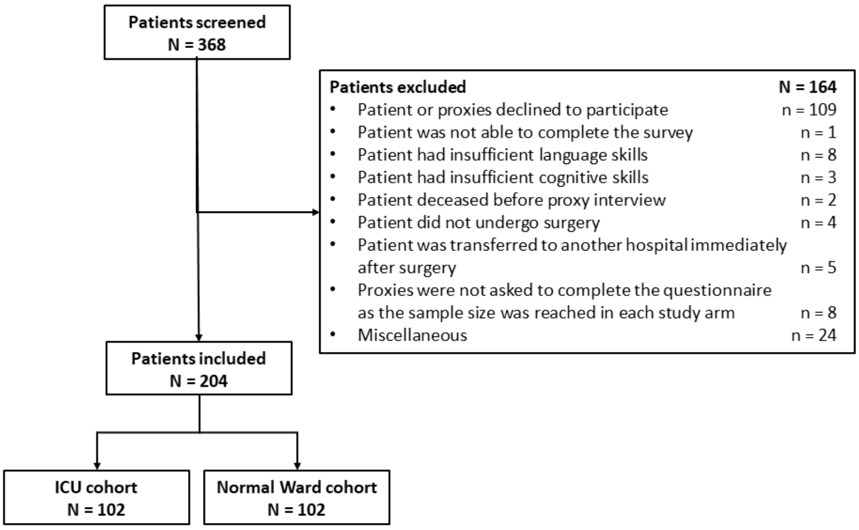

Figure S1. Study flow chart

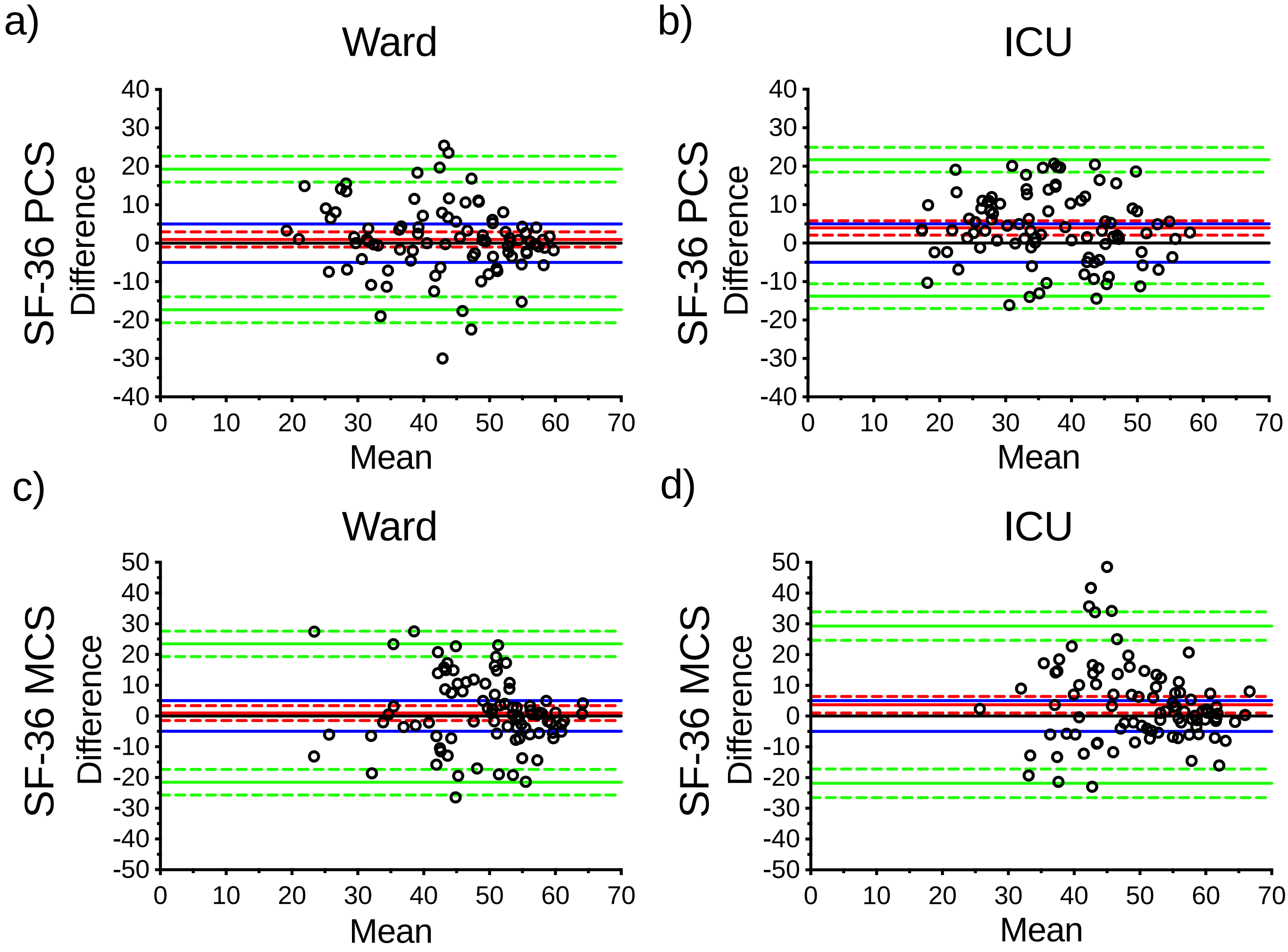

Figure S2. Reliability for regular ward and ICU in Bland-Altmann plots for the SF-36

Shown is the difference in response between patients and proxies according to the mean value of their responses. The solid red line shows the mean difference, while the dashed red lines, in conjunction with the shaded red area, indicating the corresponding 95%CI. The solid blue lines and the corresponding shaded blue area indicate the range of the minimal clinically important difference (MCID). The solid green lines show the upper and lower limit of agreement together with the grey shaded area, while the dashed green lines in conjunction with the shaded green area demonstrate the 95%CI of the limits of agreement. The SF-36 showed insufficient reliability as the 95% limits of agreement for the a)/b) PCS and c)/d) MCS for the normal ward (PCS – mean difference [95% limits of agreement]: 0.97 [-17.33 – 19.28]; MCS - mean difference [95% limits of agreement]: 0.95 [-21.56 – 23.45]), and ICU (PCS – mean difference [95% limits of agreement]: 3.93 [-13.8 – 21.67]; MCS - mean difference [95% limits of agreement]: 3.68 [-21.89 – 29.25]) patients were outside the MCID. Normal ward patients demonstrated no bias (PCS – mean difference [95% CI]: 0.97 [-0.99 – 2.94]; MCS - mean difference [95%CI]: 0.95 [-1.47 – 3.36]). ICU patients showed a significant bias towards a worse evaluation by the proxies (PCS – mean difference [95% CI]: 3.93 [2.06 – 5.81]; MCS - mean difference [95%CI]: 3.68 [0.98 – 6.38]).

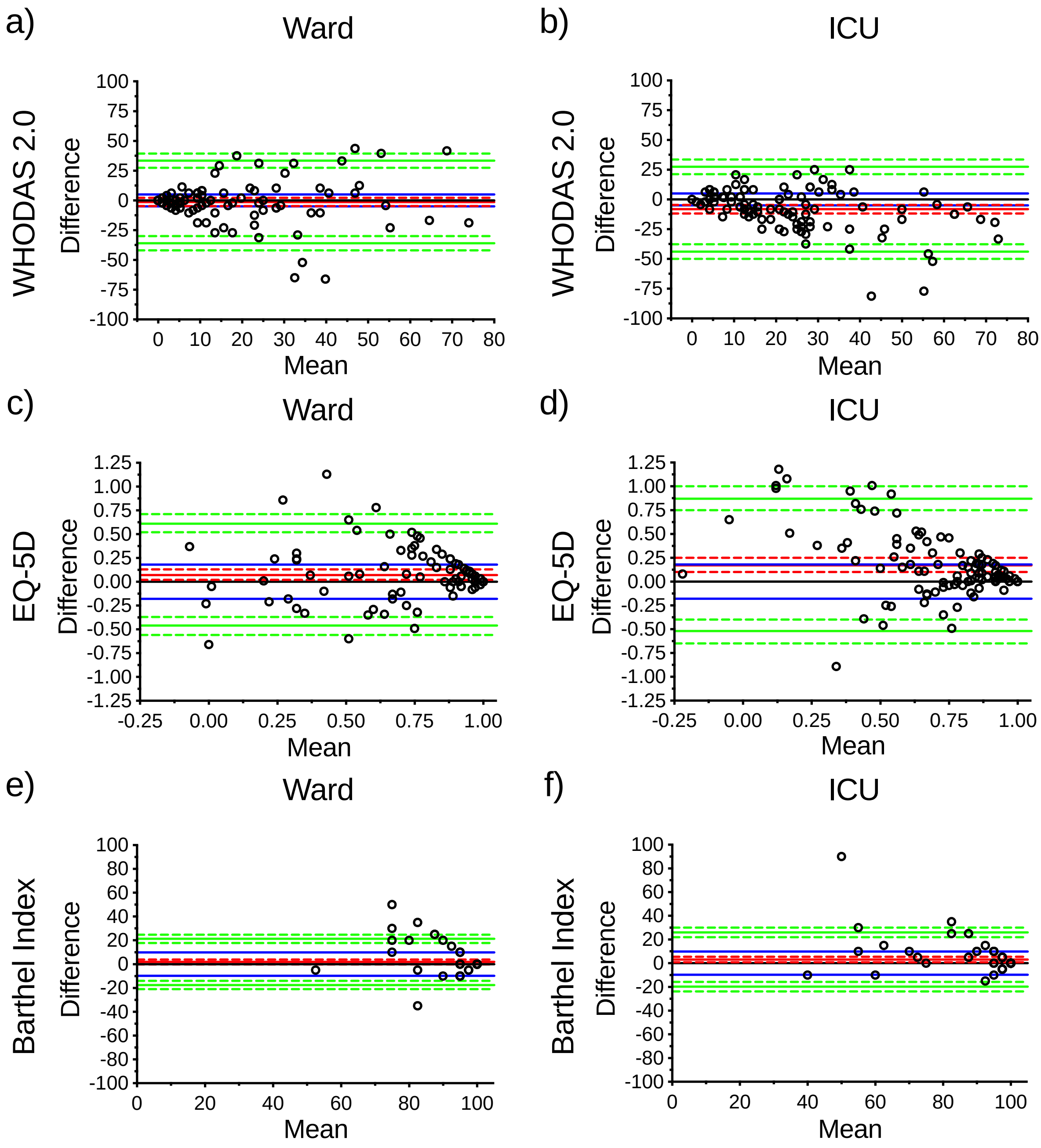

Figure S3. Reliability for regular ward and ICU in Bland-Altmann plots for WHODAS 2.0, Barthel-Index and IADL.
Shown is the difference in response between patients and proxies according to the mean value of their responses. The solid red line shows the mean difference, while the dashed red lines, in conjunction with the shaded red area, indicate the corresponding 95%CI. The solid blue lines and the corresponding shaded blue area demonstrate the range of the minimal clinically important difference (MCID). The solid green lines indicate the upper and lower limit of agreement together with the grey-shaded area. In contrast, the dashed green lines in conjunction with the shaded green area show the 95%CI of the limits of agreement. The WHODAS 2.0 demonstrated an insufficient reliability as inside the 95% limits of agreement in the Bland-Altman plot for the a) regular ward (mean difference [95% limits of agreement]: -1.33 [-36.02 – 33.37]) and b) ICU (mean difference [95% limits of agreement]: -8.27 [-43.92 – 27.37]) were outside the MCID. ICU patients showed a significant bias towards a worse evaluation by the proxies (mean difference [95%CI]: -8.27 [-11.89 - -4.69]). Normal ward patients did not show a bias (mean difference [95%CI]: -1.33 [-4.82 – 2.17]). The EQ-5D results revealed insufficient reliability as the 95% limits of agreement (normal ward - mean difference [95% limits of agreement]: 0.07 [-0.46 – 0.61]; ICU - mean difference [95% limits of agreement]: 0.17 [-0.51 – 0.87]) are outside the MCID. A significant bias toward worse evaluation by the proxies was present in the c) regular ward (mean difference [95%CI]: 0.07 [0.02 – 0.13]) and d) ICU (mean difference [95%CI]: 0.17 [0.1 – 0.25]) cohort. The Barthel Index showed insufficient reliability as the 95% limits of agreement (normal ward – mean difference [95% limits of agreement]: 1.8 [-17.6 – 21.2]; ICU – mean difference [95% limits of agreement]: 3.1 [-19.8 – 26.0]) were outside the MCID. Only f) ICU but not in e) regular wards patients, a significant bias was present (regular ward - mean difference [95%CI]: 1.8 [-0.2 – 3.9]; ICU - mean difference [95%CI]: 3.1 [0.8 – 5.4]).
